# Isoform-specific roles of AMP-activated protein kinase in cardiac physiology and pathophysiology

**DOI:** 10.3389/fcvm.2025.1638515

**Published:** 2025-08-08

**Authors:** Ani Rakoubian, Julia Khinchin, Johnathan Yarbro, Satoru Kobayashi, Qiangrong Liang

**Affiliations:** Department of Biomedical Sciences, New York Institute of Technology, College of Osteopathic Medicine, Old Westbury, NY, United States

**Keywords:** AMPK, isoform-specific, energy metabolism, mitochondria quality control, cardiac remodeling

## Abstract

AMP-activated protein kinase (AMPK) is a central regulator of cellular energy homeostasis, integrating metabolic, mitochondrial, and oxidative stress responses. In the heart, an organ with high and dynamically fluctuating energy demands, AMPK is essential for maintaining metabolic balance, particularly during conditions such as exercise, ischemia, hypertrophy, and heart failure. The AMPK complex comprises a catalytic α subunit and regulatory β and γ subunits, each with multiple isoforms (α1, α2; β1, β2; γ1, γ2, γ3) that confer tissue-specific distribution and functional specialization. This review highlights the isoform-specific roles of AMPK in the heart, emphasizing their distinct contributions to myocardial energy metabolism, contractile function, and cardiac remodeling across diverse physiological and pathological conditions.

## 1 Introduction

The heart is an energetically demanding organ that relies heavily on mitochondrial oxidative metabolism to sustain its continuous contractile function. Adenosine monophosphate-activated protein kinase (AMPK) serves as a cellular energy sensor, activated by increases in the AMP/ATP and ADP/ATP ratios, and orchestrates a metabolic shift from energy-consuming anabolic pathways to ATP-generating catabolic processes. While AMPK has long been recognized as a central regulator of cardiac metabolism, emerging research reveals that its various isoforms are not functionally redundant and may play distinct roles in cardiac physiology and disease. In the heart, different combinations of AMPK alpha (α), beta (β), and gamma (γ) subunits contribute uniquely to energy homeostasis, stress responses, and pathological processes such as hypertrophy and ischemia-reperfusion injury (1, 2). Beyond the heart, the tissue-specific distributions and functions of these isoforms exhibit across multiple organs. Isoform-specific roles have been identified in skeletal muscle, liver, and adipose tissue, where they differentially regulate glucose uptake, lipid metabolism, and mitochondrial biogenesis. These findings underscore the importance of AMPK isoform specificity in developing targeted therapies for metabolic and cardiovascular diseases. This review will highlight recent advances in our understanding of isoform-specific roles of AMPK in cardiac physiology and pathology and explore their implications for therapeutic strategies.

## 2 Overview of AMPK: structure and function

AMP-activated protein kinase (AMPK) is a heterotrimeric serine/threonine kinase that is recognized as a pivotal energy sensor within eukaryotic cells and a critical modulator of metabolic processes. The structural composition of AMPK consists of three distinct subunits: catalytic (α), scaffolding (β), and regulatory (γ) subunits, each of which is characterized by multiple isoforms (3). The α subunit contains the catalytic domain and exists in two isoforms, α1 and α2. The activation of the enzyme occurs through phosphorylation of the α subunit by upstream kinases at Thr174 (α1) or Thr172 (α2) (4–8). The beta subunit, with isoforms β1 and β2, plays a crucial role in binding to carbohydrates, including glycogen. The gamma subunit serves as the component responsible for sensing AMP, ADP, and ATP, and exhibits three isoforms in humans: γ1, γ2, and γ3 (2, 7). Each isoform is encoded by a distinct gene, where the fifth character denotes the subunit and the sixth character signifies the specific isoform: PRKAA1, PRKAA2, PRKAB1, PRKAB2, PRKAG1, PRKAG2, and PRKAG3 (2). The seven isoforms can assemble into 12 distinct heterotrimeric AMPK holoenzymes; however, only eight are expressed in cardiac tissue, as γ3 isoform is largely absent from the heart (2, 3, 7, 9, 10). A key regulatory feature of AMPK is the ADaM site (Allosteric Drug and Metabolite site), located between the α kinase domain and β subunit's carbohydrate-binding module. This site is targeted by small-molecule activators such as A-769662, salicylate, PXL770, and PF-06409577, which preferentially activate β1-containing complexes by stabilizing the holoenzyme, enhancing allosteric activation, and protecting Thr172 from dephosphorylation. The ADaM site is therefore a promising pharmacological target for modulating AMPK activity in metabolic and inflammatory diseases.

**Figure 1:**
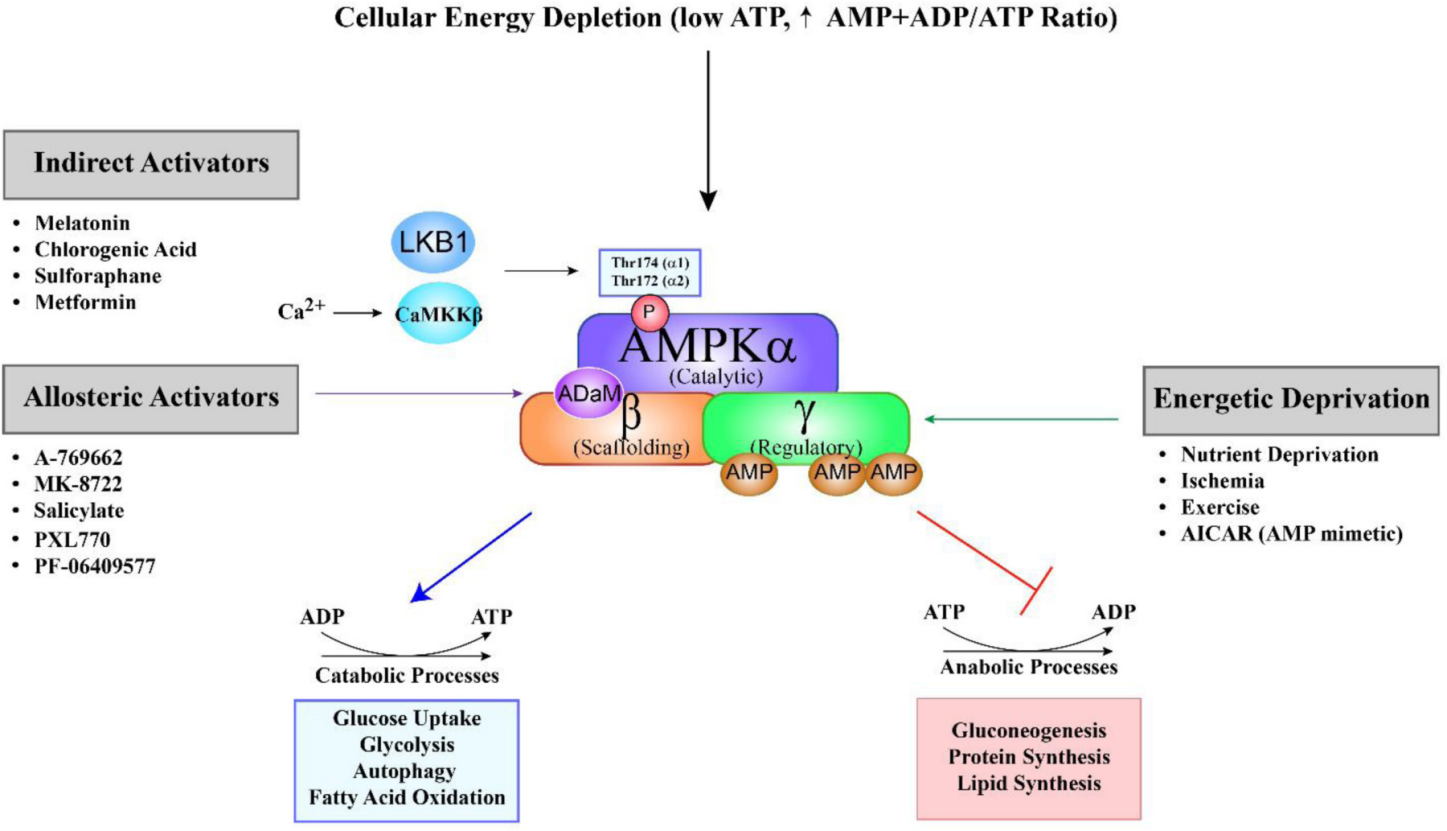
Schematic overview of AMPK activation and its metabolic effects. AMP-activated protein kinase (AMPK) is a heterotrimeric enzyme composed of a catalytic α (alpha), scaffolding β (beta), and regulatory γ (gamma) subunit. It is primarily activated by a decrease in cellular energy levels, reflected by an increased AMP + ADP to ATP ratio. Binding of AMP or ADP to regulatory sites on the γ subunit induces a conformational change that facilitates phosphorylation of the α subunit at Thr172 (α2) or Thr174 (α1) by upstream kinases, primarily LKB1 in the heart. Alternatively, CaMKKβ can activate AMPK independently of AMP/ADP in response to elevated intracellular calcium. AMPK activation can occur via three main mechanisms: (1) Energetic deprivation, triggered by conditions such as nutrient shortage, ischemia, and exercise; (2) allosteric activators, such as A-769662, MK-8722, salicylate, PXL770 and PF-06409577, which bind directly at the allosteric drug and metabolite (aDaM) site located at the interface of the alpha and beta subunits; and (3) indirect activators, including melatonin, chlorogenic acid, sulforaphane, metformin, and AICAR, which modulate upstream signaling pathways. Upon activation, AMPK shifts cellular metabolism from anabolic to catabolic pathways to restore energy balance. This includes promoting processes such as glucose uptake, glycolysis, autophagy, and fatty acid oxidation, while suppressing energy-consuming pathways like gluconeogenesis, lipid synthesis, and protein synthesis.

AMPK plays a central role in maintaining cellular energy homeostasis. It is activated in response to an increased AMP/ADP-to-ATP ratio, a hallmark of cellular energy depletion. Activation is mediated by upstream kinases such as liver kinase B1 (LKB1) and calcium/calmodulin-dependent protein kinase kinase β (CaMKKβ), which phosphorylate and activate AMPK (4–6, 8, 11). In the heart, LKB1 is the primary upstream kinase responsible for ischemia-induced activation of AMPKα2 (8, 11). In contrast, CaMKKβ is expressed at much lower levels in cardiomyocytes, and its role in cardiac AMPK regulation remains poorly understood (11, 12). Once activated, AMPK promotes energy conservation by inhibiting anabolic processes and stimulating catabolic pathways that generate ATP. Specifically, AMPK phosphorylates and inactivates key enzymes involved in gluconeogenesis, protein synthesis, and fatty acid synthesis, while simultaneously activating energy-generating pathways such as fatty acid β-oxidation to restore cellular energy balance (Figure 1) (13, 14). In turn, lipid metabolites, particularly free fatty acids (FFAs) and ceramides, modulate AMPK signaling in complex, context-dependent ways (15). AMPK activation promotes lipid oxidation and reduces steatosis, whereas ceramide accumulation, especially from saturated fats, impairs AMPK phosphorylation, drives insulin resistance, and contributes to metabolic dysfunction (15, 16). In contrast, unsaturated fats enhance AMPK activity and support metabolic health (17, 18). Despite these insights, the mechanisms by which lipids regulate AMPK remain incompletely understood, complicated by AMPK's dual role as both a regulator and a downstream target of lipid metabolism (19, 20). A deeper understanding of these interactions is critical for developing targeted therapies to restore metabolic homeostasis through the lipid-AMPK axis. Additionally, circulating factors can modulate AMPK activity in an isoform- and tissue-specific manner. For example, adiponectin preferentially activates AMPKα2 in cardiomyocytes via AdipoR1-mediated signaling, contributing to its cardioprotective effects in ischemic injury and diabetic cardiomyopathy (21). Similarly, IL-6 and other inflammation-associated cytokines activate AMPK through context-dependent mechanisms that may differentially affect the α1 and α2 isoforms (22, 23). Recent studies also show that FGF21 (24) and irisin (25) enhance cardiac AMPK activity and confer metabolic and cardioprotective benefits. Together, these findings underscore the therapeutic potential of targeting circulating regulators of AMPK in metabolic and cardiovascular diseases.

Beyond its metabolic regulation, AMPK is critically involved in cellular quality control mechanisms, including autophagy, mitochondrial fission, mitophagy, and mitochondrial biogenesis (26). During energy stress or nutrient deprivation, AMPK initiates autophagy by phosphorylating and inhibiting mammalian target of rapamycin (mTOR), a major negative regulator of autophagy (27). This inhibition activates unc-51 like autophagy activating kinase (ULK1), a kinase essential for autophagosome formation, thereby facilitating the degradation and recycling of intracellular components. AMPK has been shown to promote mitochondrial fragmentation by phosphorylating MFF at ser172 (28), facilitating the segregation and the removal of damaged segments of mitochondria by mitophagy (29). Studies using AMPKα1 knockdown mouse embryonic fibroblasts (MEFs) show that ULK1 fails to localize to mitochondria, underscoring the importance of AMPK in mitophagy (30). AMPK can also promote mitophagy via the PINK1-Parkin pathway, further implicating it in mitophagy (27). However, conflicting data from AMPKα1/α2 double knockout MEFs demonstrate that CCCP-induced colocalization of mitochondria and RFP-LC3 puncta remains unchanged compared to wild-type cells, suggesting that AMPK may not be essential for CCCP-induced mitophagy (31). Adding to the complexity, although the pan-AMPK activator MK-8722 enhances Parkin phosphorylation and promotes mitophagy, it paradoxically inhibits NIX-dependent mitophagy by inducing ULK1 phosphorylation and subsequent sequestration by 14-3-3 proteins (32). Surprisingly, mitophagy flux is also reduced in the hearts of AMPKα2-deficient mice (33). These conflicting findings underscore the need for further investigation to clarify whether AMPK's role in mitophagy is necessary, sufficient, isoform-specific, or context-dependent, and how its regulatory mechanisms vary across distinct forms of cellular stress.

AMPK also promotes mitochondrial biogenesis primarily through activation of the transcriptional coactivator PGC-1α, a central regulator of oxidative metabolism. By phosphorylating PGC-1α at Thr177 and Ser538, AMPK enhances its activity, upregulating genes involved in mitochondrial DNA replication, oxidative phosphorylation, fatty acid oxidation, and glucose uptake via GLUT4 (34). These effects are particularly important in energy-demanding tissues such as skeletal muscle, cardiac muscle, and brown adipose tissue, especially during exercise or fasting when metabolic flexibility is crucial. In contrast, conditions like obesity, hypertension, and diabetes are marked by reduced AMPK activity, leading to impaired PGC-1α function and diminished mitochondrial efficiency (34). AMPK also supports mitochondrial gene expression through epigenetic mechanisms, activating HAT1 and inhibiting DNMT1 directly through phosphorylation and indirectly via increased interaction with RBBP7 (35). In endothelial cells, pharmacological AMPK activation or shear stress triggers nucleosome remodeling and DNA demethylation, boosting expression of mitochondrial biogenesis genes including PGC-1α, TFAM, UCP2, and UCP3. These effects, also observed in AMPKα2-dependent mouse models, underscore AMPK's multifaceted role in regulating mitochondrial health (35).

Furthermore, AMPK contributes to cellular defense mechanisms by regulating transcription factors in the FOXO family (FOXO1, FOXO3, FOXO4) (36). Under metabolic stress, AMPK phosphorylates and activates FOXO proteins, enhancing their transcriptional activity. This results in upregulation of antioxidant enzymes such as superoxide dismutase (SOD) and catalase, which mitigate reactive oxygen species (ROS) and protect against oxidative damage. Through this mechanism, AMPK supports redox homeostasis, improves cellular resilience to stress, and may delay aging-related degeneration. The AMPK–FOXO axis is therefore of particular interest in therapeutic strategies targeting age-related diseases like Alzheimer's and Parkinson's disease.

Finally, AMPK plays diverse, tissue-specific roles across organ systems, largely dictated by the differential expression of its α, β, and γ subunit isoforms (10). For instance, α2 is predominant in cardiac and skeletal muscle, whereas α1 is more abundant in the liver and lungs, giving rise to distinct physiological functions. In the liver, AMPK regulates lipid metabolism and mitochondrial quality control, offering protection against steatosis and liver injury (37–39). In cancer, AMPK exhibits context- and isoform-dependent roles: it acts as a tumor suppressor by limiting anabolic growth and promoting autophagy during early tumorigenesis, but can also support tumor survival under metabolic stress once malignancy is established (40–46). In the pulmonary system, AMPK contributes to both vasodilation and hypoxic vasoconstriction, with isoform-specific effects on vascular remodeling that may inform therapies for pulmonary hypertension (47). These multifaceted roles underscore the therapeutic potential and complexity of AMPK modulation. While pan-AMPK activators like MK-8722 can improve metabolic parameters, they may also cause unintended effects such as cardiac hypertrophy (48, 49), highlighting the importance of isoform- and tissue-selective strategies.

In summary, AMPK serves as a master regulator of cellular energy status, coordinating metabolic, mitochondrial, and oxidative stress responses. Its ability to shift cells from energy-consuming to energy-generating processes makes it a pivotal mediator of both physiological homeostasis and disease progression (Figure 1). This review will focus specifically on isoform-specific functions of AMPK in cardiac physiology and pathology. For comprehensive coverage of AMPK roles in other organ systems, readers are referred to recent specialized reviews (10, 41, 47, 50, 51).

## 3 Isoform-specific functions of AMPK in cardiac physiology and pathology

In the heart, the predominant AMPK isoforms are α2, β2, and γ1/γ2, whereas the α1 isoform is more commonly expressed in non-cardiac tissues and in cardiac fibroblasts (52–54). This differential expression of isoforms is not merely structural. Rather, it plays a critical role in modulating AMPK function. The specific combination of α, β, and γ subunits determines the enzyme's activation threshold in response to metabolic stress, its subcellular distribution within cardiomyocytes, and its ability to recognize and phosphorylate distinct substrates. Furthermore, isoform composition affects AMPK's interaction with upstream kinases such as LKB1 and CaMKKβ, thereby influencing the mode and sensitivity of its activation under various physiological and pathological conditions.

### 3.1 AMPK α isoforms

Among the catalytic α subunits, α1 is expressed in cardiomyocytes (11, 55) but is more prominently found in non-myocyte populations such as endothelial cells (56) and macrophages and adipose tissue (57). Although it contributes modestly to total AMPK activity in the heart, α1 plays important roles in vascular regulation and inflammatory responses (56, 57). In contrast, α2 is the predominant isoform in cardiac myocytes, where it localizes to both the cytosol and nucleus (9, 11) and is essential for maintaining metabolic homeostasis and adapting to stress. The divergent roles of AMPKα1 and α2 in cardiac stress responses likely reflect their differing contributions to total myocardial AMPK activity, cell type-specific expression patterns (α2 in cardiomyocytes vs. α1 in non-myocytes), and subcellular localization (nuclear and cytosolic for α2 vs. cytosolic only for α1). These distinctions underscore the isoform-specific functions of AMPK in the heart, with AMPKα2 playing a dominant role in cardiomyocyte metabolism and stress adaptation. In cardiomyocyte-specific, inducible AMPKα1/α2 double KO mice, baseline heart function remained intact until old age, but stress-induced responses were impaired, including reduced exercise capacity and blunted dobutamine responsiveness. These deficits were accompanied by altered mitochondrial structure and function, decreased energy metabolism, and trends toward lower ATP and glycogen levels with age (55). While these findings underscore the essential role of AMPK in maintaining cardiac bioenergetics under stress and aging, the specific contributions of each isoform cannot be distinguished in the double knockout model.

#### 3.1.1 α1 isoform

##### 3.1.1.1. α1 in electrical remodeling

AMPKα1 plays a key role in cardiac electrical remodeling by regulating the expression and ubiquitination of connexin 43 (Cx43), a major gap junction protein critical for impulse conduction and arrhythmia susceptibility (58, 59). In wild-type (WT) mice subjected to pressure overload via transverse aortic constriction (TAC), AMPKα1 levels were elevated while Cx43 protein levels decreased, accompanied by conduction abnormalities. These effects were significantly attenuated in AMPKα1 KO mice, despite unchanged Cx43 mRNA levels, indicating a post-translational regulation. TAC also enhanced Cx43 ubiquitination in wild-type but not AMPKα1-deficient hearts. Consistently, AMPK reduced membrane Cx43 levels in Xenopus oocytes in a kinase activity–dependent manner. These findings suggest that AMPKα1 promotes Cx43 degradation via ubiquitination, contributing to gap junction remodeling and impaired electrical coupling in heart failure. While direct phosphorylation of Cx43 by AMPK has not been demonstrated, Cx43 stability is known to be regulated by MAPK family kinases (ERK, JNK, p38) (60), and AMPK can modulate MAPK signaling (61), suggesting an indirect mechanism at play. Additionally, AMPKα1 may promote Cx43 degradation by upregulating E3 ubiquitin ligases such as MuRF1, MAFbx (62), and NEDD4-2 (63).

##### 3.1.1.2. α1 in septic cardiomyopathy

AMPK activators such as AICAR and metformin have been shown to alleviate sepsis-induced cardiac dysfunction (64–66). Metformin, a first-line therapy for type 2 diabetes, activates AMPK indirectly by inhibiting mitochondrial complex I, leading to increased cellular AMP levels. AICAR, on the other hand, is metabolized into ZMP, an AMP analog that activates AMPK by binding to its γ subunit. However, both compounds also target other pathways (64, 67), so their cardioprotective effects may not be exclusively AMPK-dependent. Recent studies highlight a critical role for AMPKα1 in septic cardiomyopathy. AMPKα1 knockout mice exhibited increased cardiac microvascular permeability and higher bacterial loads following lipopolysaccharide (LPS) challenge, indicating impaired cardiac immune defense (54). In contrast, AICAR or metformin treatment in wild-type mice diminished LPS-induced vascular leakage and enhanced neutrophil-mediated pathogen clearance, but these beneficial effects were lost in AMPKα1-deficient mice. These findings indicate that the protective effects of AICAR and metformin in sepsis are mediated, at least in part, by AMPKα1, highlighting its critical immunomodulatory role in infection-induced cardiac dysfunction.

#### 3.1.2 α2 isoform

##### 3.1.2.1. α2 in ischemic heart injury

AMPKα2 plays a central role in coordinating cardiac metabolic responses under stress. It phosphorylates key regulators such as acetyl-CoA carboxylase (ACC) and TBC1D1, while also modulating transcriptional programs through coactivators like PGC-1α and MEF2. The α2-containing AMPK complexes are activated early in ischemia, promoting glucose uptake and glycolysis (68). In AMPKα2 knockout mice, the heart exhibits impaired glucose uptake and fatty acid oxidation during ischemia, indicating that α2 is essential for maintaining metabolic flexibility and adaptation (69). Importantly, AMPKα1 cannot compensate for the absence of AMPKα2, underscoring the isoform's non-redundant function in the ischemic heart. Also, pharmacological activation of AMPK with AICAR has been shown to improve left ventricular function, reduce arrhythmia incidence, and limit infarct size in isolated mouse hearts (70). However, it remains unclear whether AICAR's beneficial effects are mediated fully or partially through AMPK α2, as the study did not include AMPKα2-deficient models for confirmation.

##### 3.1.2.2. α2 in cardiac hypertrophic response

AMPK α2 attenuates cardiac hypertrophy by inhibiting key downstream effectors involved in protein synthesis, including p70 S6 kinase and eukaryotic initiation factor 4E (eIF4E) (71–73), and by reducing protein O-GlcNAcylation, a modification associated with cardiomyocyte hypertrophy (74). AMPKα2 knockout significantly worsens left ventricular hypertrophy and dysfunction in response to transverse aortic constriction, while AMPKα1 deletion has no such effect (73). Moreover, AMPKα2 deficiency markedly reduces myocardial expression of ERRα and its downstream targets, including MCAD, CPT1b, CD36, FATP1, cytochrome c oxidase subunit 3, cytochrome c, UCP3, and SOD2, under both basal and pressure overload conditions, indicating that AMPKα2 plays a dominant role over AMPKα1 in maintaining normal cardiac structure and metabolic function (73). Notably, the protective effects of Sestrin2 overexpression against pressure overload-induced hypertrophy are lost in AMPKα2-deficient hearts (71, 75). Together, these findings underscore a critical and isoform-specific role for AMPKα2 in limiting pathological cardiac remodeling in response to pressure overload.

Although AMPK is traditionally known for suppressing anabolic processes and promoting catabolism to inhibit cardiac hypertrophy (76), emerging evidence suggests it also supports selective anabolic programs in the heart that promote long-term adaptation and survival. For example, AMPK activation enhances mitochondrial biogenesis and contractile function through PGC-1α, while indirectly supporting cardiac growth by improving substrate utilization and mitochondrial efficiency, resembling adaptive responses in skeletal muscle. AMPK can also promote glycogen accumulation via glycogen synthase activation (77), a potentially maladaptive effect if unregulated. In pressure-overload models, AMPK activity increases during stress (TAC) and normalizes after unloading (De-TAC), linking its activity to the heart's anabolic capacity (78). AMPKα2, in particular, is essential for maintaining metabolic gene expression and ERRα signaling (79), underscoring its importance in cardiac anabolic processes and adaptation. Together, these findings position AMPK as a key integrator of energy stress responses and selective anabolic remodeling in the heart.

##### 3.1.2.3. α2 and cardioprotection in heart failure

AMPKα2 plays a vital role in reducing energy expenditure during heart failure. One study demonstrated that AMPKα2 deficiency leads to overexpression of cardiac-specific adenylyl cyclase 5 (AC5), which is commonly overstimulated in failing hearts, resulting in increased cAMP, energy wasting, and arrhythmias (80). In contrast, pharmacologic activation of AMPK with AICAR suppressed AC5 expression and reduced cAMP-mediated energy loss. This suggests that AMPKα2 counters the deleterious effects of chronic β-adrenergic stimulation, a hallmark of heart failure pathophysiology. Given that β-blockers are a mainstay treatment for heart failure, AMPKα2 may be integral to their cardioprotective mechanism.

##### 3.1.2.4. α2 and diabetic cardiomyopathy

AMPK plays a central role in mediating the cardioprotective effects of several antidiabetic agents, with accumulating evidence underscoring the isoform-specific importance of AMPKα2 (81). Metformin, a first-line therapy for type 2 diabetes, is associated with a reduced risk of heart failure in diabetic patients (82) and its cardioprotective effects, largely attributed to AMPK activation (81), have been validated in numerous animal and cell-based models (83–87). Importantly, metformin fails to reduce cardiac injury in OVE26 type 1 diabetic mice overexpressing a dominant-negative AMPKα2 mutant, emphasizing the essential role of this isoform in protecting against diabetic cardiomyopathy (85). Similarly, sulforaphane, a natural phytochemical, activates AMPKα2 to promote PPARα-mediated fatty acid oxidation and attenuate cardiac steatosis in diabetic models, effects that are abolished in AMPKα2-deficient mice (88), further reinforcing the isoform's role in maintaining cardiac energy metabolism.

Fibroblast growth factors FGF21 and FGF1ΔHBS also exert cardioprotective effects through AMPKα2-dependent mechanisms (24, 89). Dominant-negative AMPKα2 blunts FGF21's protection against high glucose-induced injury (24), while AMPKα2 knockdown eliminates FGF1ΔHBS-mediated protection against high glucose and palmitate-induced mitochondrial dysfunction (89). In addition to its metabolic functions, AMPKα2 also regulates mitochondria-endoplasmic reticulum (ER) interactions, with its loss leading to excessive mitochondria-associated ER membrane (MAM) formation and mitochondrial Ca²^+^ overload, defects reversed by constitutively active AMPK (90). Together, these findings highlight the central and context-specific role of AMPKα2 in preserving mitochondrial homeostasis and mediating cardioprotection under diabetic stress.

##### 3.1.2.5 α2 and autophagy/mitophagy

The molecular interplay between AMPK activation and autophagic processes in oxidative tissues remains incompletely understood (91–93). While AMPK is classically recognized for promoting autophagy initiation through ULK1 phosphorylation and mTOR inhibition, emerging evidence reveals a far more complex and context-dependent regulatory landscape. Under certain energy stress conditions, AMPK may exert inhibitory effects on autophagy and mitophagy (33, 93, 94), highlighting a dualistic role that varies by tissue type and stress context. These contradictions are particularly evident in the heart, where some studies suggest that AMPK negatively regulates mitophagy (33), while others report AMPK-dependent enhancement of mitophagy in both cardiac (95) and hepatic tissues (39). Such conflicting findings underscore a critical knowledge gap in our understanding of AMPK-autophagy crosstalk (33, 39, 93), with important implications for developing targeted therapies for metabolic and cardiovascular diseases (94, 96).

Recent work has identified AMPKα2 as a key regulator of mitophagy under cardiac stress. Specifically, AMPKα2 phosphorylates Bcl2-L-13 in response to ATP depletion, promoting mitophagy to preserve mitochondrial integrity (97). In heart failure models, a pathological shift from AMPKα2 to AMPKα1 impairs mitophagy and exacerbates mitochondrial dysfunction, whereas restoration of AMPKα2 enhances mitophagy via the PINK1/Parkin pathway and reduces ROS, effects that are lost in PINK1/Parkin-deficient systems (98). However, the role of AMPK in mitophagy is far from straightforward. For instance, mitophagy remains intact in AMPKα1/α2 double knockout MEFs treated with CCCP, suggesting alternative compensatory pathways (31). Moreover, pharmacological AMPK activation with MK-8722 paradoxically inhibits NIX-dependent mitophagy by promoting ULK1 phosphorylation and sequestration via 14-3-3 proteins (32). In yet another twist, AMPKα2 knockout hearts show enhanced mitophagy flux, evidenced by increased LC3-II accumulation on mitochondria and mito-Rosella signals (33).

Collectively, these findings suggest that AMPK's role in mitophagy is isoform-specific and highly dependent on the nature of the mitochondrial stress and the signaling context. Future studies should aim to resolve these contradictions by delineating the precise roles of AMPKα1 and AMPKα2, and by identifying the conditions under which AMPK activation is protective vs. detrimental. A clearer understanding of this complexity could unlock novel strategies to therapeutically harness AMPK-autophagy pathways in metabolic and cardiovascular diseases.

##### 3.1.2.6. α2 in doxorubicin-induced cardiotoxicity

The role of AMPKα2 in anticancer drug doxorubicin-induced cardiomyopathy remains complex and somewhat controversial. While several studies suggest that AMPK activation protects against mitochondrial damage and oxidative stress, via agents such as melatonin (99), chlorogenic acid (100), and endurance exercise (101) through the AMPK–PGC-1α pathway, other evidence points to a potentially detrimental role for AMPKα2. One study found that doxorubicin upregulates AMPKα2 via the pro-apoptotic transcription factor E2F1, promoting mitochondrial damage and cell death in H9c2 cardiomyoblasts (102). Overexpression of wild-type AMPKα2 exacerbated injury, whereas a dominant-negative mutant was protective. Notably, melatonin co-treatment suppressed AMPKα2 expression and mitigated cellular injury. Similarly, higenamine reduced DOX cardiotoxicity along with AMPK activity, while AICAR activation of AMPK abolished higenamine's protective effects (103), suggesting the detrimental nature of AMPK activation in DOX cardiotoxicity. Supporting this possibility, AMPKα2 knockout mice showed reduced cardiac injury following doxorubicin treatment (104), reinforcing the context-dependent role of AMPKα2 in mediating DOX cardiotoxicity.

In summary, AMPKα isoforms play distinct and at times opposing roles in cardiac pathophysiology. AMPKα2 is key to energy homeostasis, mitophagy, and stress resistance, while AMPKα1 appears more involved in cardiac immune responses. Conflicting data, especially in doxorubicin-induced cardiotoxicity, underscore the need for further investigation. As most studies rely on H9c2 cells, which may not fully reflect *in vivo* cardiac biology, future research should incorporate diverse models and explore the specific regulators and effectors of each isoform. Clarifying these pathways may lead to isoform-targeted therapies for various cardiac conditions, such as heart failure, diabetic cardiomyopathy, and drug-induced cardiotoxicity.

### 3.2 AMPK β isoforms

The β subunits of AMPK serve as scaffolding proteins and contain a glycogen-binding domain, playing essential roles in cardiac development and metabolic regulation. They mediate interactions with phosphatases and influence AMPK activation kinetics. The heart expresses both β1 and β2 isoforms, with β2 being predominant (105). Deletion of both AMPK β isoforms using the muscle creatine kinase (MCK) promoter–driven Cre results in impaired systolic and diastolic function at baseline (106), highlighting their essential role in maintaining cardiac physiology. Interestingly, β1/β2 double KO mice exhibit more severe cardiac dysfunction than α1/α2 double KOs (55), which may be attributed to the loss of AMPK signaling in skeletal muscle or during development, as MCK-Cre is active in both skeletal and cardiac muscle from developmental stages. Despite sharing 71% sequence homology, β1 and β2 differ significantly at the N-terminus (105), contributing to distinct posttranslational modifications, substrate affinities, and subcellular localizations. These differences result in isoform-specific functions in cardiac metabolism and gene regulation (107). Notably, β2 modulates AMPK localization to glycogen particles, with significant impact on glucose and glycogen metabolism (108).

#### 3.2.1 β1 isoform

##### 3.2.1.1. AMPKβ1 and metabolic maturation of cardiomyocytes

Compared to β2, AMPKβ1 has a more specialized role in regulating cardiac metabolism. It is expressed in liver, heart, kidneys, and lungs (10), and contributes to the metabolic maturation of cardiomyocytes. Specifically, AMPKβ1 facilitates the metabolic shift from glycolysis to fatty acid oxidation, a transition that enhances ATP yield and reflects cardiomyocyte maturation (109). This adaptation supports the high energy demands of the contracting heart and its ability to respond to physiological stress.

#### 3.2.2 β2 isoform

##### 3.2.2.1. AMPKβ2 and cardiac lineage differentiation

β2 is especially critical during early cardiac lineage specification. Studies using human-induced pluripotent stem cells (hiPSCs) have shown that AMPKβ2 is pivotal for cardiomyocyte differentiation and maturation (107). In murine models, deletion of PRKAB2 (encoding AMPKβ2) resulted in a complete lack of cardiac differentiation, whereas deletion of AMPKβ1 led to impaired cardiomyocyte function despite preserved differentiation (107). Furthermore, AMPKβ2 deficiency disrupts mesoderm and endoderm differentiation while promoting ectodermal lineage expansion, underscoring its specific role in cardiogenesis. This aligns with its broad expression in metabolically active tissues such as cardiac muscle, skeletal muscle, liver, and adipose tissue (10).

#### 3.2.3 Pharmacological activation of β isoforms

Pharmacological activation of specific AMPK isoforms has been investigated to enhance cardiomyocyte function. A-769662, a potent allosteric activator of AMPKβ1-containing complexes (110), binds directly to the ADaM site at the interface of the α and β subunits, enhances phosphorylation at Ser108 (109), and exhibits significantly greater efficacy than AICAR in promoting vasodilation of resistance arteries (67, 111). Unlike A-769662, AICAR binds to the γ subunit of AMPK to activate the enzyme, and its use is limited by off-target effects and poor clinical tolerability (64, 67). A-769662 has also been shown to boost mitochondrial biogenesis and function, evidenced by increased oxygen consumption, ATP production, and metabolic gene expression (109). A-769662 exerts anti-inflammatory effects in models of acute heart and lung injury, supporting its potential as a cardioprotective agent (112). However, A-769662 also inhibits Na^+^/K^+^-ATPase independently of AMPK, raising concerns about its specificity (113). Similarly, salicylate activates AMPK heterotrimers containing β1 but not β2 subunits (114) through an AMP-independent mechanism (115). Whether salicylate offers greater specificity than A-769662 remains uncertain.

More recently, two small-molecule AMPK activators, PXL770 and PF-06409577, have been identified, both exhibiting a preferential bias toward complexes containing the β1 subunit. PXL770 has advanced to clinical evaluation and demonstrated favorable safety and efficacy in a phase 2a trial for non-alcoholic fatty liver disease (NAFLD), showing improvements in metabolic parameters and suggesting potential for broader application in type 2 diabetes and non-alcoholic steatohepatitis (NASH) (116). Similarly, PF-06409577 has shown therapeutic promise in preclinical models, correcting NAFLD and lowering cholesterol in rodents and primates (117). Notably, PF-06409577 also exerts anti-inflammatory and anti-fibrotic effects, including reduced macrophage-driven inflammation and atherosclerosis (118). Together, these agents highlight the therapeutic potential of β1-selective direct AMPK activators in treating metabolic and inflammatory diseases.

Recent studies have identified AMPK-stabilizing compounds such as PF-739 and MK-8722 that enhance AMPK activity by stabilizing the α2β2γ1 complex, leading to improved glucose uptake and favorable metabolic effects in preclinical models. PF-739 selectively activates β2-containing AMPK complexes and shows efficacy in lowering blood glucose without major adverse effects (119). MK-8722, a broad-spectrum AMPK activator, demonstrates potent metabolic benefits but also induces reversible cardiac hypertrophy (48), likely due to non-selective activation of AMPKγ2, a subunit linked to hypertrophic signaling. These findings highlight both the therapeutic potential of AMPK activation and the need for isoform- and tissue-selective modulators to minimize off-target effects. Developing such selective agents is a key priority for advancing AMPK-based therapies for metabolic and cardiovascular diseases.

### 3.3 AMPK γ isoforms

The AMPK γ subunit exists in three isoforms, γ1, γ2, and γ3, encoded by PRKAG1, PRKAG2, and PRKAG3, respectively. As the energy-sensing component of the AMPK complex, the γ subunit monitors cellular energy status by detecting changes in the AMP/ADP to ATP ratio (7). Each isoform contributes uniquely to AMPK regulation and function, thereby influencing cardiac metabolism and disease processes. Among these, γ1and γ2 are the predominant isoforms expressed in the heart, while γ3 is minimally expressed in cardiac tissue with poorly defined functions (10, 120).

#### 3.3.1 γ1 isoform

The γ1 subunit is the most abundant in cardiac tissue and is involved in general energy regulation. It is crucial for maintaining basal AMPK activity and responding to metabolic stress by promoting catabolic pathways that generate ATP (120). In rodent models of myocardial ischemia, AMPK γ1 accounts for approximately 70% of total AMPK activity and is the primary driver of Thr172 phosphorylation on the catalytic α subunit in cardiomyocytes (121, 122).

#### 3.3.2 γ2 isoform

The γ2 isoform plays a central role in a distinct form of familial hypertrophic cardiomyopathy known as *PRKAG2 cardiac syndrome* (123–126), which results from overexpression or mutation of PRKAG2 (127). This syndrome is characterized by glycogen accumulation in cardiomyocytes, ventricular preexcitation, and arrhythmias due to aberrant conduction (127), highlighting the unique, non-redundant role of γ2 in cardiac electrical and metabolic homeostasis (128). Specific PRKAG2 mutations, such as K475E and N488I, impair AMP sensing and lead to hyperactivation of downstream signaling, particularly mTOR (127). In a study by Zhuo et al. (127), adenoviral overexpression of PRKAG2 in H9C2 cells led to increased AMPK levels, cardiac hypertrophy, enhanced mTOR signaling, and excessive glycogen storage. These effects were mitigated by treatment with the β-blocker metoprolol, which reduced AMPK activity and Akt/mTOR phosphorylation, suggesting a mechanistic link between PRKAG2 overexpression and mTOR-driven hypertrophy (127).

Interestingly, PRKAG2-driven hypertrophy appears to preferentially activate the α2 isoform of AMPK over α1 (129, 130). While AMPKα2 is involved in mitochondrial glucose uptake in cardiomyocytes, AMPKα1 has been linked to myocardial fibrosis by promoting myofibroblast activation following ischemia (130). This isoform-specific distinction supports the hypothesis that PRKAG2-mediated hypertrophy may stem, at least in part, from enhanced glycogen accumulation within cardiomyocyte mitochondria (130). However, genetic inhibition of glucose-6-phosphate-stimulated glycogen synthase, which blocks glycogen storage, eliminated the ventricular preexcitation but did not affect the excessive cardiac growth in N488I mutant mice (131). Notably, this hypertrophic response was prevented by rapamycin, an mTOR inhibitor, suggesting that the Akt/mTOR pathway, rather than glycogen accumulation, plays a dominant role in mediating cardiac hypertrophy associated with the N488I PRKAG2 mutation (131).

As a key metabolic sensor, AMPK is activated during exercise in response to energy depletion, primarily through AMP binding to its γ subunit, which promotes autophosphorylation of the α subunit at Thr172 (121). Among the catalytic isoforms, AMPKα2 is activated more rapidly and at lower exercise intensities than AMPKα1, which requires more prolonged or intense activity for activation (121). This indicates that AMPKα2 plays a predominant role in modulating energy homeostasis during exercise. However, in individuals with PRKAG2 cardiac syndrome, where AMPKα2 signaling may be dysregulated, exercise-induced activation of this isoform could potentially exacerbate hypertrophic signaling (121, 130). Therefore, exercise regimens should be carefully considered in this population.

AMPKγ2 has been shown to confer cardioprotection in the setting of ischemia/reperfusion injury (9). In addition to its role in energy sensing, AMPKγ isoforms exhibit distinct subcellular localization: γ1 is predominantly cytoskeletal, while γ2 localizes to mitotic nuclei. A study by Cao et al. (9) demonstrated that cardiac-specific activation of AMPKγ2 in transgenic mice suppressed rRNA and ribosomal protein synthesis, thereby reducing endoplasmic reticulum (ER) stress. In contrast, AMPKγ2 knockout mice displayed heightened ER stress and were more vulnerable to ischemic injury. Under stress conditions, nuclear translocation of AMPKγ2, rather than γ1, leads to formation and activation of the AMPKα2/β1/γ2 complex (9). This γ2-driven suppression of ribosome biogenesis and mitigation of ER oxidative stress highlight AMPKγ2 as a potential therapeutic target for I/R injury. However, given its association with pathological hypertrophy in PRKAG2 cardiac syndrome, further investigation is needed to clarify the dual roles of AMPKγ2 in both cardioprotection and disease pathogenesis.

## 4 Summary

AMPK is a master metabolic regulator and energy sensor, playing critical roles across various tissues, including the heart. In cardiomyocytes, AMPK's cardioprotective function is executed through its heterotrimeric composition of α, β, and γ subunits, each existing in multiple isoforms with distinct roles. These isoforms orchestrate key cellular processes such as autophagy, mitophagy, mitochondrial biogenesis, and differentiation, which are essential in maintaining myocardial energy balance and cellular survival. AMPK activation has been strongly associated with cardioprotection during pathological cardiac events, particularly in ischemia-reperfusion injury, heart failure, and metabolic diseases like diabetic cardiomyopathy. However, its therapeutic efficacy depends heavily on understanding the isoform-specific functions and expression patterns in cardiac tissue. The isoform-specific functions of cardiac AMPK are summarized in Table 1.

**Table 1:** A summary of AMPK isoform-specific functions in the heart.

Subunit/isoform	Gene	Cardiac expression/localization	Major cardiac roles	Key pathological/clinical associations	Other notable features
Alpha-1 (α1)	PRKAA1	Low in cardiomyocytes; higher in cardiac fibroblasts, endothelial cells, non-myocytes (52–54)	- Regulates cardiac electrical remodeling (connexin 43, gap junctions) (58, 59). - Modulates cardiac immune response (sepsis) (133).	- Electrical remodeling, arrhythmia susceptibility (58, 59) - Septic cardiomyopathy (immune defense, microvascular permeability) (133)	Cannot compensate for α2 loss in metabolic adaptation; more prominent in non-cardiac tissues (lung, liver) (10)
Alpha-2 (α2)	PRKAA2	Predominant in cardiomyocytes (cytosol and nucleus) (11)	- Coordinates metabolic adaptation to stress (ischemia, exercise) (69) - Promotes glucose uptake, glycolysis, fatty acid oxidation (68). - Inhibits hypertrophy (protein synthesis pathways) (71, 72) - Regulates mitophagy and mitochondrial quality (97, 98) - Modulates cAMP signaling in heart failure (80).	- Essential for ischemic adaptation and metabolic flexibility (69) - Attenuates cardiac hypertrophy (71, 74, 75) - Required for mitophagy-mediated cardioprotection (97) - Cardioprotective in diabetic cardiomyopathy and heart failure (80) - Controversial role in doxorubicin cardiotoxicity	Activated by metformin, sulforaphane, AICAR (70); cannot be compensated by α1 in heart; rapidly activated by exercise (70)
Beta-1 (β1)	PRKAB1	Expressed in heart (less than β2), kidney, lung (10)	- Regulates metabolic maturation (glycolysis to fatty acid oxidation) (109) - Supports metabolic adaptation during cardiac growth/stress	- Impaired function leads to defective metabolic maturation in cardiomyocytes (109)	Targeted by A-769662 (β1-specific activator) (109, 110) and salicylate (114, 115); involved in anti-inflammatory effects (112); less critical for differentiation (107)
Beta-2 (β2)	PRKAB2	Predominant in cardiomyocytes; also in skeletal muscle, liver, adipose (10)	- Essential for cardiac lineage specification and differentiation (107) - Modulates AMPK localization to glycogen (108) - Impacts glucose/glycogen metabolism (108)	- Deletion blocks cardiac differentiation and disrupts mesoderm/endoderm specification (107)	Required for early cardiac development; defines gene signature in cardiac lineage specification (107)
Gamma-1 (γ1)	PRKAG1	Most abundant γ isoform in heart; cytoskeletal localization (10)	- Maintains basal AMPK activity (120) - Major driver of AMPK activation (Thr172 phosphorylation) in ischemia (122)	- Accounts for ∼70% of AMPK activity during myocardial ischemia (120)	General energy regulation; responding to metabolic stress (121)
Gamma-2 (γ2)	PRKAG2	Highly expressed in heart (mitotic nuclei) (9); forms α2/β1/γ2 complex (9)	- Central in PRKAG2 cardiac syndrome (familial hypertrophic cardiomyopathy) (124–130) - Regulates glycogen storage, electrical conduction, and mTOR signaling (127) - Enhances stress adaptation via ER stress and ribosomal synthesis regulation (9)	- PRKAG2 mutations cause glycogen storage, hypertrophy, arrhythmia, preexcitation (124–130) - Cardioprotection in ischemia/reperfusion injury (9)	Overactivation causes mTOR-driven hypertrophy (127); nuclear translocation under stress (9); activates α2 more than α1 (9)
Gamma-3 (γ3)	PRKAG3	Minimally expressed in heart	No established role in cardiac tissue	Poorly defined	Major γ isoform in skeletal muscle (10)

The α subunit isoforms, AMPKα1 and AMPKα2, perform distinct roles in cardiomyocytes. AMPKα1 primarily contributes to cardiac electrical remodeling and plays a key role in the pathophysiology of septic cardiomyopathy. In contrast, AMPKα2 enhances energy efficiency during heart failure by modulating intracellular cAMP levels and promoting mitophagy, thereby facilitating the removal of dysfunctional mitochondria. Activation of AMPKα2 by drugs like metformin, AICAR, and sulforaphane has been shown to reduce apoptosis and increase fatty acid oxidation, enhancing myocardial survival. However, contrasting findings in doxorubicin-induced cardiotoxicity, where AMPKα2's role is ambiguous, highlight the need for more controlled, isoform-specific studies, ideally using standardized human cell models to reduce variability across experimental systems.

The β subunits, AMPKβ1 and AMPKβ2, also diverge in function. AMPKβ2 is essential during early cardiac development, guiding mesodermal differentiation into mature cardiomyocytes. Without it, proper structural and metabolic maturation is impaired. In contrast, AMPKβ1 facilitates the metabolic shift from glycolysis to fatty acid oxidation during cardiac growth and stress. This adaptation is key to energy-efficient function, particularly under conditions like ischemia. A-769662, a β1-specific activator, has demonstrated anti-inflammatory potential during myocardial infarction, though concerns remain about off-target effects, such as Na^+^/K^+^-ATPase inhibition. Similarly, PF-06409577 exhibits both anti-inflammatory and anti-fibrotic properties, including suppression of macrophage-mediated inflammation and attenuation of atherosclerosis. Collectively, these findings underscore the therapeutic promise of β1-selective AMPK activators in the treatment of metabolic and inflammatory disorders.

The γ subunit, particularly AMPKγ2, functions as an energy sensor, responding to AMP/ATP ratios to fine-tune cellular energy usage. AMPKγ2 enhances cardiac resilience during ischemic stress by reducing ER stress and modulating ribosomal activity. Yet, mutations in PRKAG2, the gene encoding γ2, lead to chronic overactivation, resulting in glycogen accumulation, pathological hypertrophy, and conduction abnormalities. These outcomes, collectively described as PRKAG2 cardiac syndrome, underscore the dual-edged nature of AMPKγ2 activity, highlighting the challenge of harnessing its benefits without triggering adverse remodeling.

## 5 Future perspectives

While the therapeutic potential of AMPK modulation is compelling, realizing its clinical application requires a more nuanced understanding of isoform-specific functions and their context-dependent effects. Future drug development should prioritize isoform-selective AMPK activators to reduce off-target consequences. For example, MK-8722, though effective in activating AMPKβ2 and enhancing glucose metabolism, also activates AMPKγ2, inadvertently promoting cardiac hypertrophy. Developing activators with improved specificity and well-defined therapeutic windows will be essential for leveraging AMPK's benefits without inducing unintended side effects (132).

These pharmacologic strategies should be guided by a deeper understanding of isoform-specific AMPK signaling, particularly its interactions with pathways such as Akt/mTOR and transcriptional coactivators like PGC-1α, which regulate mitochondrial biogenesis and fatty acid oxidation. Such precision targeting is essential for effectively treating complex cardiac conditions including heart failure, diabetic cardiomyopathy, and ischemic injury.

In parallel, lifestyle interventions, such as exercise and dietary modification, provide non-pharmacologic avenues for AMPK activation. Exercise, in particular, selectively activates AMPKα2, promoting autophagy and enhancing mitochondrial quality control in heart failure models. When combined with pharmacologic therapies, these approaches may yield synergistic benefits for both prevention and management of cardiac disease.

In summary, unlocking the full therapeutic potential of AMPK in cardiovascular medicine will require a multifaceted approach that includes:

1. Elucidating isoform-specific roles across the α, β, and γ subunits,
2. Mapping downstream signaling pathways in cardiac-specific contexts,
3. Developing isoform-selective activators that minimize off-target effects, and
4. Standardizing preclinical models, particularly by using human cardiomyocytes, to enhance translational relevance.
